# Estimated Breeding Values for Canine Hip Dysplasia Radiographic Traits in a Cohort of Australian German Shepherd Dogs

**DOI:** 10.1371/journal.pone.0077470

**Published:** 2013-10-29

**Authors:** Bethany J. Wilson, Frank W. Nicholas, John W. James, Claire M. Wade, Peter C. Thomson

**Affiliations:** 1 Faculty of Veterinary Science, The University of Sydney, Sydney, New South Wales, Australia; CSIRO, Australia

## Abstract

Canine hip dysplasia (CHD) is a serious and common musculoskeletal disease of pedigree dogs and therefore represents both an important welfare concern and an imperative breeding priority. The typical heritability estimates for radiographic CHD traits suggest that the accuracy of breeding dog selection could be substantially improved by the use of estimated breeding values (EBVs) in place of selection based on phenotypes of individuals. The British Veterinary Association/Kennel Club scoring method is a complex measure composed of nine bilateral ordinal traits, intended to evaluate both early and late dysplastic changes. However, the ordinal nature of the traits may represent a technical challenge for calculation of EBVs using linear methods. The purpose of the current study was to calculate EBVs of British Veterinary Association/Kennel Club traits in the Australian population of German Shepherd Dogs, using linear (both as individual traits and a summed phenotype), binary and ordinal methods to determine the optimal method for EBV calculation. Ordinal EBVs correlated well with linear EBVs (*r* = 0.90–0.99) and somewhat well with EBVs for the sum of the individual traits (*r* = 0.58–0.92). Correlation of ordinal and binary EBVs varied widely (*r* = 0.24–0.99) depending on the trait and cut-point considered. The ordinal EBVs have increased accuracy (0.48–0.69) of selection compared with accuracies from individual phenotype-based selection (0.40–0.52). Despite the high correlations between linear and ordinal EBVs, the underlying relationship between EBVs calculated by the two methods was not always linear, leading us to suggest that ordinal models should be used wherever possible. As the population of German Shepherd Dogs which was studied was purportedly under selection for the traits studied, we examined the EBVs for evidence of a genetic trend in these traits and found substantial genetic improvement over time. This study suggests the use of ordinal EBVs could increase the rate of genetic improvement in this population.

## Introduction

Canine hip dysplasia (CHD) has been reported to be the one of the most prevalent musculoskeletal disorders of the dog [1]. It is a developmental disorder of the coxo-femoral joint in which excessive looseness and malcongruency of the joint structures leads in many cases to debilitating osteoarthritis in one or both hips [2]–[4]. The mode of inheritance of CHD is multifactorial, meaning that many genes and many non-genetic factors contribute to variation in the CHD phenotype. There is some evidence for genes that have a relatively large effect [5]–[10]. Several control schemes have been established worldwide for the control of CHD. Most schemes involve determination of phenotypes from radiographic examination of the hips. The specific CHD phenotype measured, the positioning and sedation of the dog for radiography, and the age at which dogs are eligible for scoring, vary among schemes.

Selection against CHD based upon a phenotype, such as a score determined from a radiograph, is based upon the premise that the score of the radiograph is a least partly heritable. The *extent* to which the radiographic score is heritable (its “heritability”) relates to the extent to which a candidate’s superiority/inferiority is caused by allele superiority/inferiority and may therefore be passed on to offspring. For traits such as CHD, environmental factors are very important influences on pathology, and therefore only a sizeable minority of the variation in radiographic scores is explained by allelic variation (i.e., the heritability is moderate).

The calculation of estimated breeding values (EBVs) is an established technology in production animals for improving the effectiveness of selection on any trait. Using mixed-model equations, an EBV can be calculated for a particular trait based not only on the candidate’s phenotypic score but all relevant information such as scores from relatives (who share alleles with the candidate to a predictable extent), scores from related traits which may be due to the actions of some of the same genes, and, when available, molecular information regarding some of the many germane genes. Most published estimates of the heritability of CHD radiographic phenotypes have been within the range 0.2 to 0.6 [11]. Selection on traits with heritabilities in this range can most definitely benefit from the use of EBVs. However, few breeding schemes have utilised EBVs for the control of CHD, although many authors have recommended EBV use [12]–[15] and have demonstrated the feasibility of EBVs for a variety of CHD scoring schemes [6], [14], [16]–[18]. The Seeing Eye Inc began using EBVs in 1995 and soon observed substantial reductions in radiographic signs of CHD [6].

The method of CHD scoring that has been most extensively used in Australia is based on the British Veterinary Association (BVA)/Kennel Club (KC) scheme and, following the UK in Australia, by the AVA/ANKC (Australian Veterinary Association/Australian National Kennel Club) scheme. The PennHip method [3] has also been endorsed more recently in Australia. The BVA/KC Scheme is based on a radiograph taken with hips in extension. Nine traits for each hip (referred to in this paper as British Veterinary Association Hip Traits – BVAHTs) are assessed against an ordinal categorical scale in which each category is labelled with a number between 0 (normal) and 6 (most extreme change) or, for one trait (Caudal Acetabular Edge – CaAE), between 0 and 5. Previous work on these phenotypes in Australian German Shepherd Dogs (GSDs) has suggested that the right and left scores for each phenotype are determined by the same set of genes, with bilateral differences determined by other factors [19], [20].

It has also been shown from the same Australian GSD data sets that the nine traits can usefully be grouped on the basis of the magnitude of their phenotypic variance into the more variable “Group 1” traits of Norberg Angle (NORB), Subluxation (SUBL) and Cranial Acetabular Edge (CrAE), and the substantially less variable “Group 2” traits of Dorsal Acetabular Edge (DAE), Cranial Effective Acetabular Rim (CrEAR), Acetabular Fossa (AF), CaAE, Femoral Head and Neck Exostosis (FHNE) and Femoral Head Remodelling (FHR) [11]. Lewis et al. [17] proposed the same grouping in a study of BVAHTs of Labrador Retrievers from the United Kingdom, based on the reality that Group 1 traits tend to reflect morphological malformations and Group 2 traits reflect mainly pathological signs of osteoarthritis. Given the relative youth of animals scored for BVAHTs (median age of 19 months) in the Australian GSD data set, and the tendency of osteoarthritis to increase with age, it is perhaps not surprising that more extreme phenotypes are seen among the Group 1 traits which may measure earlier changes. Typically, selection using BVAHTs has been undertaken by summing each of a dog’s 18 scores (category labels) and using this number as a performance phenotype which can be used to compare animals relative to each other and also to an “average” performance phenotype for the breed.

Calculation of EBVs for BVAHTs is complicated by the underlying ordinal nature [21] of the phenotypes. The extent to which it is valid to assume that joint category labels represent a ratio-scale of numbers which are evenly spaced, and similar between BVAHTs, and therefore suitable to be added, is unproven. In considering this issue, Lewis et al. [22], Lewis et al. [17], Wood et al. [23], Wood et al. [24] and Wood et al. [25], elected to proceed as if there were integrity in the linearity and ratio scalarity of the category numbers. However, the extent of correctness of doing so could potentially vary between breeds and between breed groups in different countries. One study of EBVs for an ordinal hip dysplasia phenotype found considerable individual differences in EBV rankings, depending upon whether a linear method, or a method accounting for the nonlinear nature of the data, was used [26].

The aim of this paper is to explore the potential for EBV-based selection in Australian GSDs. We investigate the extent to which the calculation of EBVs results in an appreciable increase in the accuracy of genetic merit estimation compared with phenotypic evaluation alone. We compare EBVs derived from ordinal logistic regression (the method which best reflects the underlying nature of the data) and from simpler methods of analysis. Also, the calculation of EBVs for a cohort with dates of birth spanning decades allows us to assess whether any favourable genetic trend has occurred and, from this, to infer whether any effective selection pressure has been exerted in this dog population.

## Materials and Methods

### Data

Two sources of CHD data were used in this study, namely data accumulated by Dr Malcolm Willis in the United Kingdom from records collected within the Australian Veterinary Association/Australian National Kennel Council (AVA/ANKC) canine hip and elbow dysplasia scheme (CHEDS) and the records of radiologists sent to him privately; and data supplied by the German Shepherd Dog Council of Australia (GSDCA) hip dysplasia breed scheme. Pedigree information regarding Australian GSDs held by the ANKC was supplied with permission of the GSDCA by Dogs NSW. All data sets included all data available electronically at the time at which the records were obtained. Details of the data set are available in [11]. The data set contains the animal’s name, pedigree information, year of birth, age at radiographic study, sex and scores for each of the 18 BVAHTs. The final data set analysed in this study comprised records from 13,124 (8,793 female, 4,331 male) GSDs born in Australia between 1976 and 2006. Completeness of the data set was investigated by matching scores against the pedigree file for Australian-born GSDs and is reported in [11]. A detailed description of the nature of the data, including a distribution of scores, is also provided in [11].

### Models Used to Calculate EBVs

EBVs were obtained from the Best Linear Unbiased Prediction (BLUP) or BLUP-like estimates produced by the “stand alone” version of ASReml 3 (VSN Intl., Hemel Hempstead UK) when analysing the BVAHT data using a series of different models, namely (1) an ordinal logistic model that treats the BVAHT data in a multi-threshold approach; (2) a series of binary logistic models that fits a separate threshold model at each BVAHT score; and (3) a standard linear mixed model on the individual and summed BVAHT data, ignoring the ordinal nature of the data. A more complete discussion of these models is available in [11]. Differences between right and left scores for each BVAHT in this data set arise almost solely due to environmental and non-additive genetic causes [19], and are therefore considered repeated measures in the following models. Note that inherent in all the EBV calculations modelled below is the estimation of all relevant variances (and hence heritability estimates) from the same data from which EBVs were determined.

1. Ordinal (multi-threshold) analysis

This model considers two scores (left and right) from each of *n* dogs. For a single observation in the data set, the model has the following form,
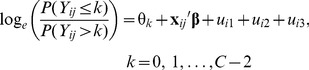
(1)where *Y_ij_* is the BVAHT score of the *i* ^th^ dog (*i* = 1, …, *n*) on the *j* ^th^ side (*j* = 1, 2), *C* is the number of points on the ordinal scale (*C* = 7 for all BVAHT except CaAE where *C* = 6). For each cut-point, there is a separate “intercept” (θ*_k_*), with the constraint that θ_0_< θ_1_< …<θ*_C_*_ –2_. **β** is a vector of *p* levels of fixed effects related to the vector of explanatory variables, **x** *_ij_*. The random effects for this model are *u_i_*_1_, a term for the dog’s breeding value; *u_i_*_2_, a term for the permanent environment effect of the dog linking left and right hand scores together; and *u_i_*_3_, a litter effect. This form of ordinal logistic regression is known as the proportional odds model [27]. Note that the EBV is taken as the BLUP of the *u_i_*_1_ with its sign swapped to facilitate the interpretation that lower EBVs indicate lower scores.

1. Binary (logistic) analysis

In addition to the multi-threshold ordinal analysis of the nine BVAHTs, binary modelling was undertaken at each possible cut-point, i.e. at each interval (on a scale) at which a threshold can be used to divide dogs into two classes: normal and affected. To accommodate the binary nature of these data, a logistic generalised linear mixed model (GLMM) was fitted to the data.

The form of the GLMM is
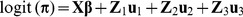
(2)where 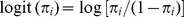, and π*_i_* is the probability that dog *i* has a score at or below the cut-point. 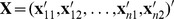 is a 2*n*×*p* matrix of predictor variables and **β** is a vector of *p* levels for the same fixed effects as (1) and **Z** *_r_* are indicator matrices relating to random effects **u** *_r_*, *r* = 1, 2, 3 as given in (1). A separate logistic GLMM was fitted for each BVAHT × cut-point combination. As in the ordinal analysis, the signs of the BLUPs of the *u_i_*_1_ were swapped in order to obtain the EBVs.

1. Linear mixed models (LMM)

In this analysis, each of the nine BVAHTs was modelled by an LMM using the awarded score as the trait. Scores were transformed logarithmically to attempt to correct positive skew in the distribution of the scores. While improved, substantial skew remained for many traits. Stronger transformations, while possible, were not attempted. The model was of the form:
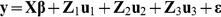
(3)


The vector **y** represents a vector of 2*n* = 26,248 log-transformed hip scores, and **ε** is a 2*n*×1 vector of random residual effects, where 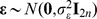.

In all three models, fixed effects incorporating **β** include the sex of the dog (male or female), the variable age of the dog in months at the time of radiographic study and the year in which the dog was born. The random effects **u**_1_ are “animal model” additive genetic effects. The animal model is fitted by calculation of the numerator relationship matrix (NRM), a matrix of additive genetic relationships which contains information about the flow of genes through the population and information enabling accounting for inbreeding. The model also assumes that 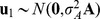 where 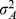 is the additive genetic variance, **A** is the NRM; and also that 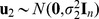 and 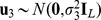.

In addition to the analysis of individual BVAHTs, total hip scores (THS) were obtained by addition of the 18 BVAHT scores for each dog. This has been the standard trait used for selection in the CHEDS and in the GSDCA scheme. The scores were again logarithmically transformed. A linear mixed model was then fitted to the THS data of the form:
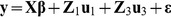
(4)where the vector **y** now represents a vector of *n* = 13,124 log-transformed THS observations, and **X** does not include a term for left versus right hip. The model does not include a permanent environment effect for the dog as there was only one result per dog, but does include a litter effect (**u**_3_).

1. Accuracy of EBVs

The accuracy of an EBV was calculated using the formula
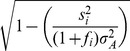
(5)where *s_i_* ^2^ is the standard error for the EBV (*u_i_*_1_) for individual *i*, *f_i_* is the inbreeding coefficient of individual *i* derived from the NRM and 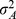 is the additive genetic variance component [28].

1. Correlation of EBV method

Pearson’s correlation coefficients were calculated between the EBVs for each of the BVAHTs using R statistical software [29].

1. Genetic trend

In order to evaluate the extent of genetic improvement over time in the BVAHT’s units, the EBVs obtained in the ordinal analysis (1) were converted into values expressed on the original BVAHT scale and the change in phenotype scale proportions were graphed over time. While it was possible to express genetic improvement more conventionally as a line graph of average EBVs over time, it is not intuitive to relate such a graph back to the ordinal BVAHT scale. These converted values were calculated as follows. From (1):
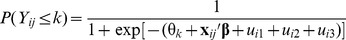
(6)


The mean fixed effects (**x** *_ij_*) were weighted by the proportions in the original data, the breeding value effect (*u_i_*_1_) was evaluated at the mean for the particular year of birth, the permanent environment (*u_i_*_2_) and the litter effects (*u_i_*_2_) were evaluated at their means, i.e. zero. These means were then substituted into (6), allowing the plotting of the different ordinal threshold *k* by year of birth from 1980–2005.

## Results

### Ordinal EBVs

**Figure 1:**
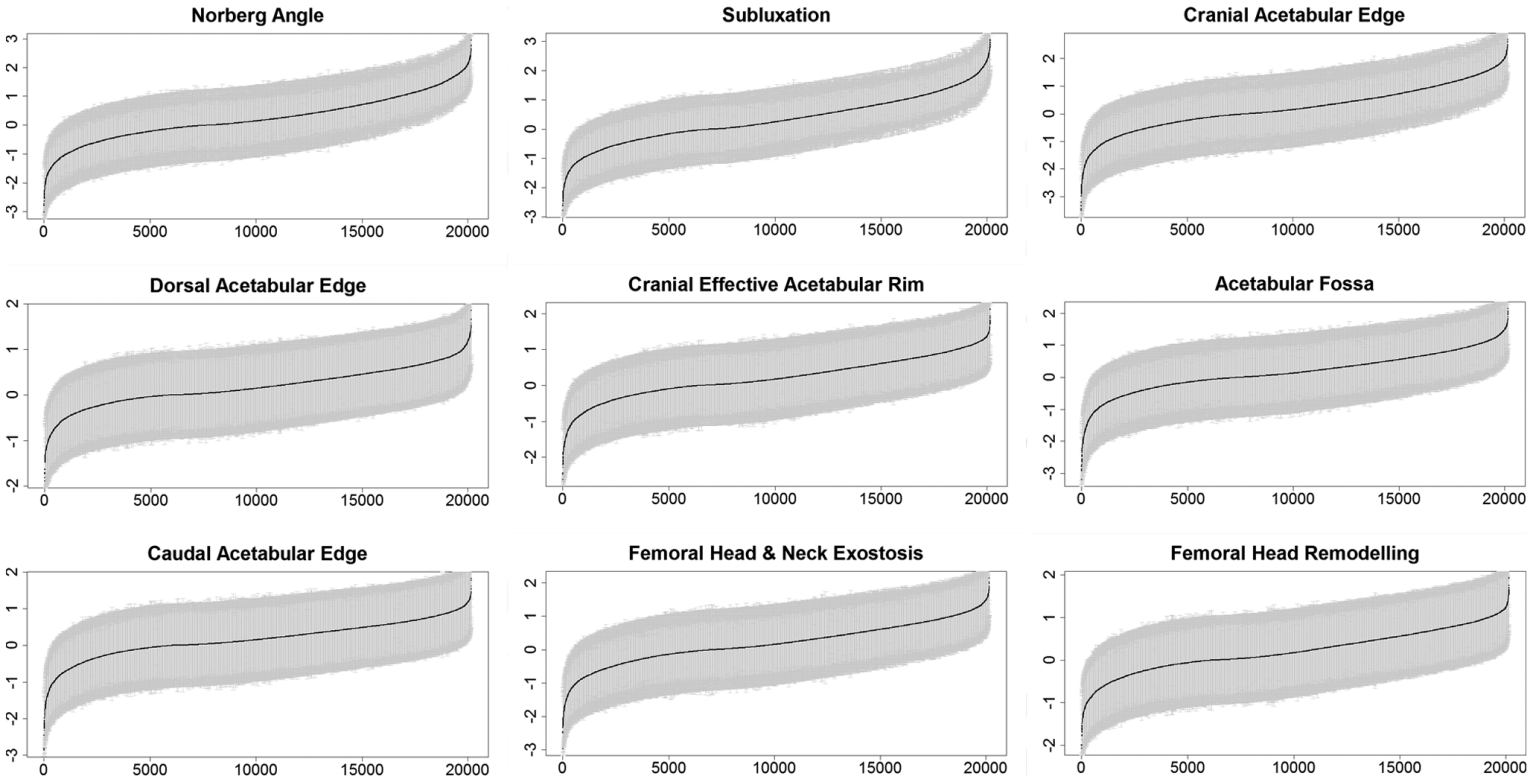
Ordinal estimated breeding values and standard errors from a multi-threshold mixed-model analysis of BVAHTs of a cohort of Australian German Shepherd Dogs.

Because this methodology most correctly reflects the underlying nature of the data, these EBVs were considered the standard for comparison. Ordinal EBVs with error bars are presented in Figure 1. The graphs show that these EBVs display a suitably normal distribution.

Average EBVs for dogs with hip dysplasia records are shown in Table 1. Average EBVs for dogs without records, included in the pedigree as ancestors to show pedigree structure, were near zero and are also shown. As expected, averages of standard errors of EBVs were higher for animals without phenotypic records than for animals with records, although some dogs without records but with many descendants and relatives with records produced relatively small standard errors, down to 0.26–0.46 depending on the trait in question.

**Table 1:** Average estimated breeding values (EBV) and average standard errors (SE) for Australian German Shepherds with and without a set of BVA/KC hip phenotypes available.

	Dogs with hip records		Dogs included as ancestors only	
Trait	EBV	SE(EBV)	EBV	SE(EBV)
NORB	−0.37	0.92	0.01	1.16
SUBL	−0.51	0.85	0.03	1.09
CrAE	−0.35	0.99	0.03	1.25
DAE	−0.27	0.84	−0.01	0.92
CrEAR	−0.33	0.93	0.00	1.07
AF	−0.27	0.99	0.03	1.17
CaAE	−0.26	0.99	0.02	1.10
FHNE	−0.30	0.96	0.00	1.14
FHR	−0.33	0.89	0.00	1.00

NORB = Norberg Angle; SUBL = Subluxation; CrAE = Cranial Acetabular Edge; DAE = Dorsal Acetabular Edge; CrEAR = Cranial Effective Acetabular Rim; AF = Acetabular Fossa; CaAE = Caudal Acetabular Edge; FHNE = Femoral Head and Neck Exostosis; FHR = Femoral Head Remodelling.

**Figure 2:**
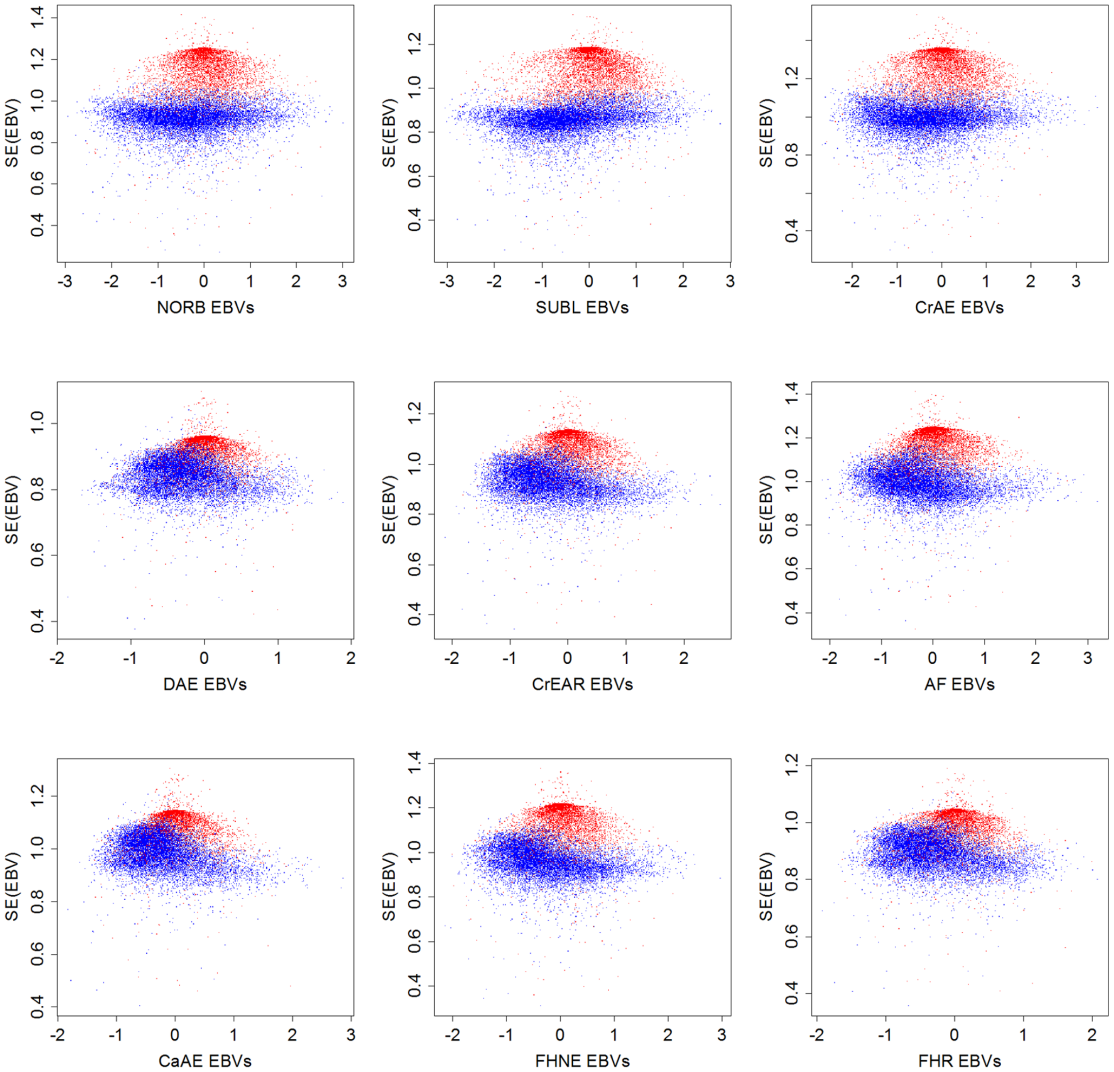
Relationship between ordinal EBVs and their standard errors. Animals with phenotypic scores are shown in blue, animals without phenotypic scores are shown in red, NORB = Norberg Angle, SUBL = Subluxation, CrAE = Cranial Acetabular Edge, DAE = Dorsal Acetabular Edge, CrEAR = Cranial Effective Acetabular Rim, AF = Acetabular Fossa, CaAE = Caudal Acetabular Edge, FHNE = Femoral Head and Neck Exostosis, FHR = Femoral Head Remodelling.

Relationships between the ordinal EBVs and their standard errors can be seen for each hip trait in Figure 2. The primary reason for the two-banded appearance appears to be the presence of animals with data records and animals included only as ancestors, as the vast majority of data points in the lower band of each graph represent animals with data records, while animals appearing as ancestors only cluster in each upper band.

### Accuracies

**Figure 3:**
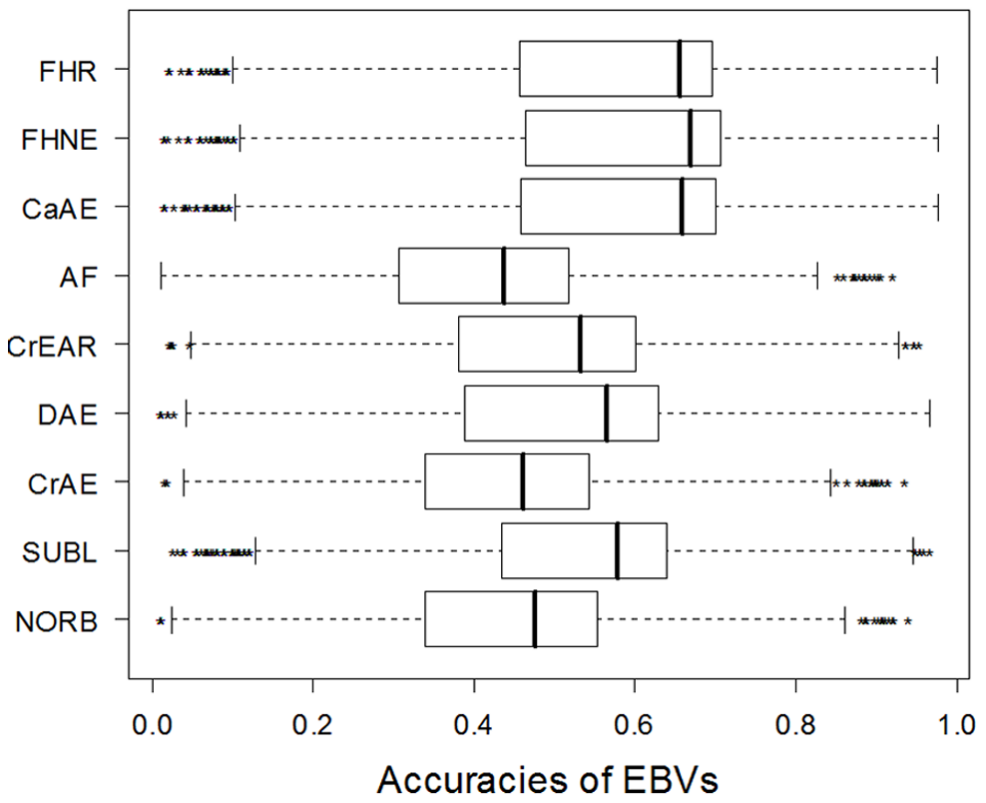
Boxplots of EBV accuracies for BVAHTs for a cohort of Australian German Shepherd Dogs. NORB = Norberg Angle, SUBL = Subluxation, CrAE = Cranial Acetabular Edge, DAE = Dorsal Acetabular Edge, CrEAR = Cranial Effective Acetabular Rim, AF = Acetabular Fossa, CaAE = Caudal Acetabular Edge, FHNE = Femoral Head and Neck Exostosis, FHR = Femoral Head Remodelling.

The accuracy of an EBV is determined in part from the standard error associated with the EBV. The range and distribution of accuracies for all ordinal EBVs are shown in Figure 3. Although the standard errors around each of the ordinal EBVs appear quite large in comparison to the variation in the relative magnitude of the EBVs (Figure 1), it is evident that for some animals a high degree of accuracy was attainable, especially in the more phenotypically variable traits.

The accuracy of breeding decisions based on a breeding candidate’s phenotype alone can be calculated as the square root of the heritability [30]. Table 2 shows the greater accuracy of ordinal EBV selection compared with selection based solely on individual phenotype. As demonstrated in Wilson et al. [11] BVAHTs have a moderate heritability, and in this heritability range, including additional information such as phenotypes of relatives by use of EBVs is expected to improve accuracy to a useful extent. In this analysis, for animals with their own phenotype available, the average accuracy for EBV selection is substantially greater than that expected for selection by phenotype alone, and accuracies are above zero for many animals without phenotypic records.

**Table 2:** Accuracies of phenotype-only selection compared with average accuracies from EBVs obtained by the ordinal model (See Table 1 for abbreviations).

	With own phenotype		Without own phenotype	
	Fromphenotypeonly	From EBV	FromPhenotypeonly	From EBV
**NORB**	0.51	0.68	0	0.32
**SUBL**	0.50	0.69	0	0.33
**CrAE**	0.52	0.69	0	0.32
**DAE**	0.40	0.48	0	0.23
**CrEAR**	0.49	0.57	0	0.27
**AF**	0.50	0.60	0	0.28
**CaAE**	0.50	0.50	0	0.26
**FHNE**	0.49	0.61	0	0.30
**FHR**	0.46	0.52	0	0.25

Accuracy with which phenotype reflects the breeding value is obtained as the square root of the heritability. Accuracy of phenotype selection is by definition 0 (or no better than chance) for animals without BVA/KC phenotype data available.

### Correlation between EBVs for Different BVAHTs

**Figure 4:**
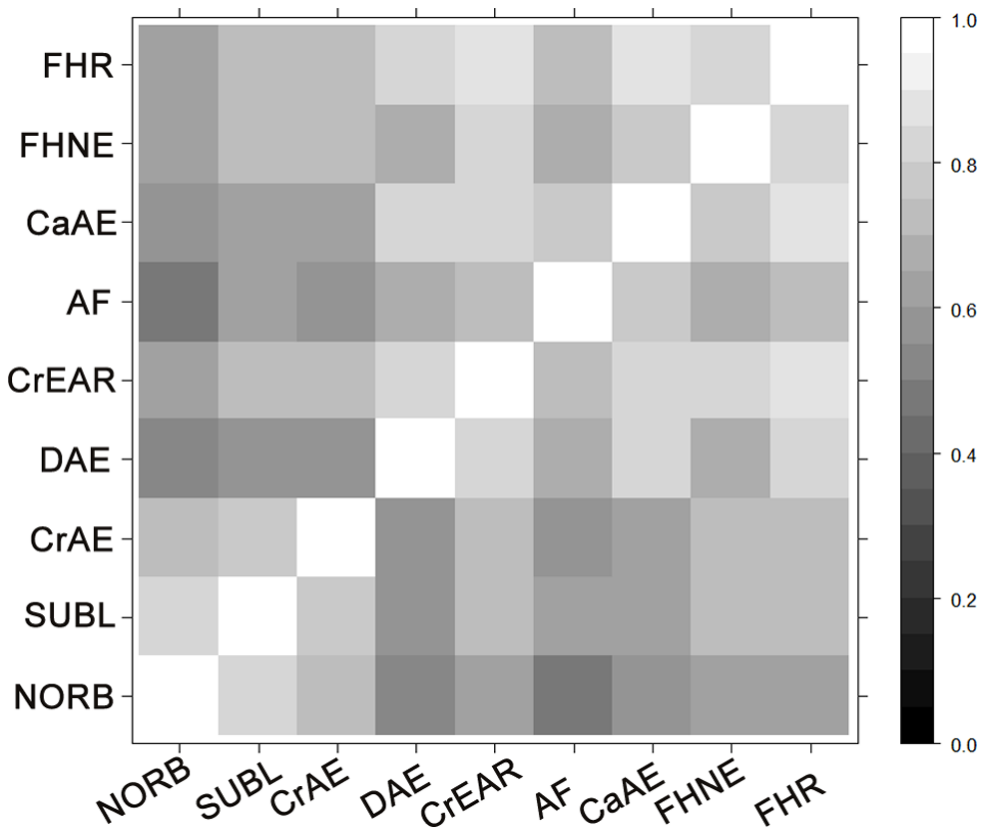
Correlation between EBVs for nine BVAHTs calculated by ordinal logistic regression. NORB = Norberg Angle, SUBL = Subluxation, CrAE = Cranial Acetabular Edge, DAE = Dorsal Acetabular Edge, CrEAR = Cranial Effective Acetabular Rim, AF = Acetabular Fossa, CaAE = Caudal Acetabular Edge, FHNE = Femoral Head and Neck Exostosis, FHR = Femoral Head Remodelling.

The correlations between the EBVs for the nine ordinal traits are illustrated in Figure 4. Correlations vary between 0.49 and 0.86. Correlation between EBVs is expected to approximate, but underestimate, the genetic correlation, as genetic correlation is the correlation between the true breeding values which the EBV estimates, and EBVs regress toward the mean. These findings are therefore suggestive that there is, unsurprisingly, substantial genetic correlation between these BVAHTs. Genetic correlations of between 0.58 and 0.91 (in absolute value) for different hip phenotypes were reported by Zhang et al. (2009).

The average correlation among Group 1 trait EBVs (NORB, SUBL, CrRAE) is 0.77 and among Group 2 trait EBVs (DAE, CrEAR, AF, CaAE, FHNE and FHR) is also 0.77. The average correlation between Group 1 and Group 2 trait EBVs is 0.64. The somewhat higher intra-group correlation to intergroup correlation may offer some evidence towards our hypothesis that Group 1 and Group 2 traits are measuring two somewhat different underlying traits, potentially our hypothesised laxity (early CHD) trait and osteoarthritic (late CHD) trait. That there is a genetic correlation between the two groups is not surprising, as joint laxity is considered to be causative of the osteoarthritic changes. The lower than 1 intergroup EBV correlations suggest that the intergroup genetic correlation may also be less than 1 and therefore, for a given degree of laxity, there would be animals with varying genetic tendencies towards osteoarthritis. It may be advantageous to identify animals with a decreased genetic tendency toward osteoarthritis for a given laxity, either for molecular studies or else when considering the role which they should play in a breeding program.

### Binary EBVs

Binary EBVs were calculated for every dog at each cut-point for each trait and compared to ordinal EBVs for each trait using a Pearson correlation coefficient (Table 3).

**Table 3:** Correlation between EBVs calculated by binary logistic regression with cut-points introduced between different scores and EBVs calculated by the ordinal model (See Table 1 for abbreviations).

	Cut-point between scores					
	0 and 1	1 and 2	2 and 3	3 and 4	4 and 5	5 and 6
**NORB**	0.88	0.93	0.83	0.70	0.62	0.47
**SUBL**	0.72	0.93	0.90	0.82	0.55	0.39
**CrAE**	0.86	0.90	0.77	0.63	0.51	0.44
**DAE**	0.99	0.92	0.86	0.78	0.72	0.65
**CrEAR**	0.98	0.94	0.84	0.77	0.67	0.63
**AF**	0.97	0.83	0.72	0.63	0.54	0.48
**CaAE**	0.99	0.92	0.84	0.75	0.63	n/a
**FHNE**	0.97	0.87	0.78	0.65	0.61	0.24
**FHR**	0.99	0.96	0.88	0.75	0.67	0.33

Generally, binary EBVs agreed best with ordinal EBVs when the cut-point divided the data more evenly (data not shown).

### Linear Mixed Model EBVs

**Figure 5:**
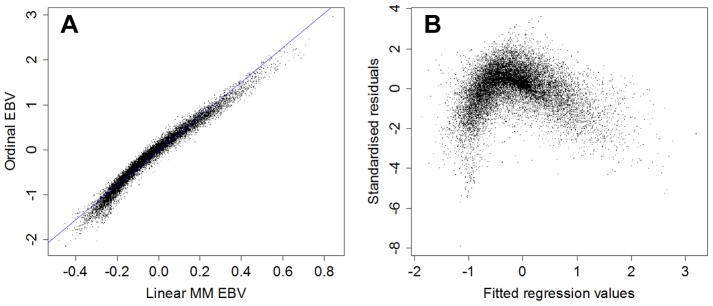
A- Linear EBVs (x) vs ordinal EBVs (y) for Femoral Head and Neck Exostosis (FHNE; a Group 2 trait) and B- fitted regression values(x) vs standardised residuals(y). Note that although the correlation is high (0.96) and the relationship on the left appears approximately linear; it is clear from the graph on right that the assumptions of a simple linear regression are not met.

EBVs were also calculated under the assumption that the score scale was linear. Generally, comparing linear and ordinal EBVs for each of the hip phenotypes revealed a relatively linear relationship for Group 1 traits, but a substantially nonlinear relationship for Group 2 traits. As correlations were generally quite high (see Table 4), attempts were made to fit a simple linear regression model to the two sets of EBVs. Plots of the fitted values, versus the residuals, however, indicate that the model is not a good fit, particularly for Group 2 traits, and that the relationship between linear EBVs and the ordinal EBVs is not sufficiently linear to allow linear EBVs to serve as an adequate proxy (e.g. see Figure 5 and see Figure S1).

**Table 4:** Pearson correlation between EBVs using a linear mixed model (LMM) and an ordinal model for nine BVAHTs in Australian German Shepherd Dogs (See Table 1 for abbreviations).

Trait	Correlation
**NORB**	0.99
**SUBL**	0.98
**CrAE**	0.99
**DAE**	0.94
**CrEAR**	0.97
**AF**	0.98
**CaAE**	0.90
**FHNE**	0.98
**FHR**	0.95

### Linear Mixed Model EBVs from Summed BVAHTs

Correlation and regression parameter estimates for a comparison of the ordinal EBVs with summed EBVs (based on the summed total of the 18 scores) are presented in Table 5. The correlation between EBVs ranges from moderate (0.58) to high (0.92), and appears higher for NORB, SUBL and CrAE than for the remaining traits.

**Table 5:** Correlation of estimated breeding values calculated by a linear mixed model on summed BVAHTs with EBVs calculated by an ordinal model on individual BVA/KC hip phenotypes in a cohort of Australian German Shepherd Dogs (See Table 1 for abbreviations).

	Correlation
**NORB**	0.86
**SUBL**	0.92
**CrAE**	0.85
**DAE**	0.58
**CrEAR**	0.71
**AF**	0.61
**CaAE**	0.62
**FHNE**	0.75
**FHR**	0.72

### Genetic Trends in BVAHTs

**Figure 6:**
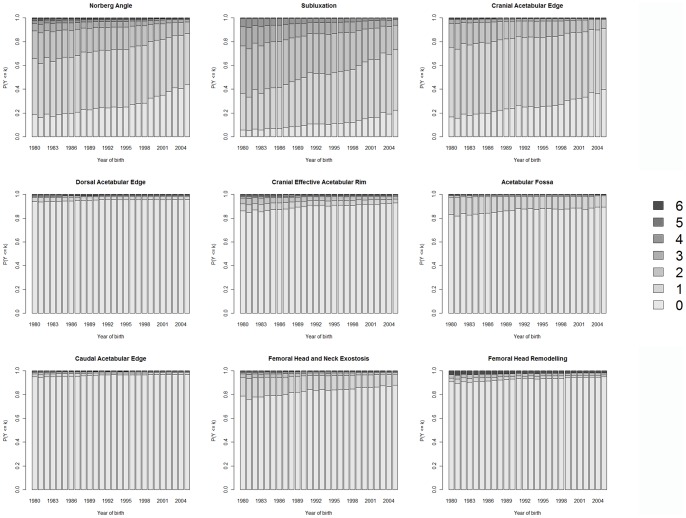
Trend of EBVs by year of birth expressed in terms of their effects on the observed BVAHT scale for a cohort of Australian German Shepherd Dogs born from 1980–2005. Proprtions for each score are derived from an ordinal model analysis of observed BVAHT scores. Increasing proportions in lower scores represent a genetic improvement over time.

The genetic trend in each of the nine BVAHTs, expressed in their original scales, is shown in Figure 6. A trend of genetic improvement is evident, with greater genetic improvement among laxity/morphology related “Group 1” traits than in osteoarthrtitis-related “Group 2” traits.

## Discussion

The main aim of this paper was to explore the feasibility and utility of ordinal mixed model-based estimated breeding values for Australian GSDs as a method for increasing the accuracy with which the genetic merit of potential parents can be assessed in relation to BVAHTs. The ordinal EBVs showed a sufficiently normal distribution and resulted in, as expected, a substantially more accurate assessment of genetic merit for BVAHTs than phenotypic selection alone.

The ultimate aim of selection for the control of CHD should be to reduce the lifetime pain, distress and loss of function experienced by dogs as a consequence of CHD. While this is the most relevant goal to the welfare of the dog and would therefore represent the best phenotype on which to base selection, it is also currently not possible to quantify. Furthermore, even if it could be quantified, this cannot occur until the dog’s end of life. Unable to select on the true selection goal, breeding programs for CHD are forced to select another, *indirect*, phenotype which, it is hoped, is genetically correlated with this true selection goal. Unfortunately, despite the intricacy of the BVAHT-based phenotypes, there is a regrettable paucity of information about how each phenotype relates to animal welfare. A study by Malm et al. [31] considered the relationship between insurance claims for CHD-related morbidity and mortality. While the low availability and utilisation of pet insurance in Australia makes such a study implausible for this population, more information on the relationship between each BVAHT phenotype and end-of-life outcomes would be invaluable to rationally allocate selection pressure between the BVAHTs as part of an overall selection program.

Most populations of pedigree dogs have multiple genetic disorders, or undesirable heritable traits which should be addressed. However, the amount of selection pressure which is available is limited. Therefore, it is helpful to consider selection pressure as a limited and precious resource that should be apportioned and expended wisely in the pursuit of improved canine welfare. Welfare-based breeding objectives for each breed should be carefully considered and prioritised as objectively as possible [32], [33], and improvements in breeding value accuracy should be sought conscientiously to avoid needless squandering of selection pressure on inaccuracy. Canine hip dysplasia has the potential to cause considerable pain, and substantially limit quality of life, and treatment can be incomplete, ineffective, expensive and prone to significant complications. It is therefore likely to be among welfare-based breeding priorities in most canine populations in which clinical disease occurs with any frequency, including Australian GSDs. The improved utilisation of selection pressure which EBVs can provide is highly desirable in this and similar populations.

The nine BVAHT ordinal EBVs correlated moderately to highly with each other, which suggests that they can be successfully combined into a simpler selection index while retaining much of the information. There were substantially higher EBV correlations within the groupings potentially indicated by the range of phenotypic variation (Group 1: NORB, SUBL, CrAE, and Group 2: DAE, CrEAR, AF, CaAE, FHNE, FHR) than between the two groups, suggesting that these groups may be partially differentially measuring something representing the animal’s inherent hip laxity (measured to a greater extent by Group 1) and its tendency to develop osteoarthritic change in response to this laxity (measured to a greater extent by Group 2). In studying genetic correlations between aggregated Group 1 and Group 2 traits, Lewis et al. [17] found genetic correlations of 0.89–0.92 depending on the method of data transformation, suggesting that the traits were affected by many, but not all of the same gene loci. The correlations between the ordinal EBVs reported here were somewhat lower than this. While it is likely that ordinal EBV correlations from the current study are underestimating the true genetic correlation, it is worth noting that the genetic correlations reported by Lewis et al. [17] were obtained using a linear mixed model which does not necessarily model the ordinal traits optimally. Also, there is no reason to assume that genetic correlations between the two trait groups would be identical in two different populations, even if there were no differences in the way which the two populations were scored, which itself would be unlikely. In this case the two populations were different breeds (Labrador Retrievers vs GSDs). Generally speaking however, geographically isolated populations with limited gene flow between them should have their CHD genetic parameters evaluated independently, even if of the same breed as they may have very different gene frequencies and have been under very different selection pressures, both historically and currently.

Both laxity and osteoarthritic potential are relevant to the welfare of dogs with CHD. There may be clinical pain and abnormal gait associated with hip laxity even prior to the development of osteoarthritis and the degree of laxity has been clearly related to the amount of osteoarthritis. Lewis et al. [17] opined that it “may be argued that there is a moral obligation in selecting against the cause of malformation rather than the severity of the consequences”. We would argue that there is a moral imperative to select in the manner which we hope will lead to the least suffering due to hip dysplasia regardless of whether that suffering is due to the malformation itself, or to the osteoarthritic response. Selection for dogs that exhibit a relatively mild osteoarthritic response to joint laxity (and against dogs who exhibit relatively more severe osteoarthritis) may be a means of reducing suffering in concert with selecting to reduce laxity itself. Research which explores the correlation between the various BVAHT scores and improves our understanding of suffering due to hip dysplasia is, in any case, urgently required.

A considerable advantage of EBV-based selection over simple phenotype-only selection is the ability to obtain EBVs from animals for which there are no phenotypic data available. This could be especially useful in the comparison of puppies from different litters which are too young to be radiographically assessed for canine hip dysplasia. Currently there would be no way of selecting between puppies too young for assessment from the same litter, as the information from relatives available for each would be identical and the EBVs would therefore be equal. However, the potential for EBVs to be enhanced by inclusion of information from molecular markers in the future could potentially allow such selection [34]. EBVs can also allow selection between animals with the same phenotypic score by including information from relatives, and potentially also molecular markers, with evidence-based weightings.

An additional aim of the paper was to compare EBVs based on an ordinal mixed model to those based on linear and binary mixed models. While methodologically simpler, use of a linear model for BVAHTs assumes that the numbered classes are equidistantly spaced on a non-arbitrary underlying scale, which cannot be justified by the available information. In fact, our finding (see Figure 5) is that even when linearly- and ordinally-derived EBVs are highly correlated, the relationship between them is not linear. Despite strong correlations between the linear and ordinal EBVs, and equally strong or stronger rank correlations (data not reported), the non-linearity of the association demonstrates that the linear and ordinal EBVs calculated here are not interchangeable. Even though the two methods would rank animals similarly, the relative genetic merits of breeding animals would be less accurately understood using linear EBVs. It is possible that stronger transformations of scores to obtain a more normal distribution may have improved the adequacy of the fit of the model and its resultant linear EBVs relative to the ordinal EBVs. It was felt, however, that stronger transformations would have substantially complicated the approachability of the analysis for non-specialists. As this approachability represents the major potential advantage of linear-method EBVs over ordinal-method EBVs, overall the use of ordinal EBVs appears preferable. In this study, the computation time for ordinal models were slightly longer than for linear models, but computation times remained very manageable on a standard desktop computer.

Analysing ordinal data such as BVAHTs requires specification of assumptions such as the proportional odds assumption as used in the above regression model, although other forms of ordinal models are available [27]. In contrast, the EBVs based on binary models are methodologically simpler than ordinal EBVs, as they do not require such assumptions. Details on the pathological findings associated with BVAHT scores are described thoroughly elsewhere [20]. Earlier work [11] demonstrated heritable phenotypic variation at almost all BVAHT cut-points. However, given the relative paucity of data which link BVAHTs to clinical outcomes, selection of the appropriate cut-point for the binary scale would be necessarily somewhat arbitrary. Additionally, any attempt to force a quantitatively variable trait into a binary outcome (such as “affected” vs “unaffected”), while clinically convenient, results in substantial loss of information, such that an animal which is only a borderline fail appears the same as the animal with the worst possible phenotype. Therefore, while binary analysis may be the only method which strictly meets all the assumptions of analysis, this rectitude comes with a severe loss of information. For the present breed population, the authors believe that the ordinal-based EBVs are the best balance between methodological suitability and complexity, and recommend them in preference to the linear EBVs based on tenuous assumptions and the binary EBVs which necessitate detrimental discarding of considerable phenotypic detail.

Concerns have been raised about the potential for EBVs to be biased because of the inclusion of non-random offspring data in EBV calculation [15], [35]. Offspring are only likely to be radiographed for BVAHT evaluation if they are of interest as a breeding candidate and this could result in offspring with detectable CHD signs being radiographed less frequently. Additionally, submission of films taken for BVAHT evaluation is voluntary. Paster et al. [36] have documented a tendency for more favourable films to be submitted to the Orthopedic Foundation for Animals for CHD evaluation at a higher rate than less favourable films, suggesting that breeders and veterinarians may decide not to submit some unfavourable films once taken. Stock et al. [35] have suggested that breeders who own popular sires may be more likely to withhold favourable films to prevent poor scores adversely affecting the EBVs of their stock [35]. Because of these biases a culture of submission of all films should be encouraged by breed societies for the mutual benefit of all members in accurately assessing the genetic merit of breeding candidates. Similarly, incorporation of molecular information which, as noted by Stock et al. [35], should be less prone to submission bias than phenotypic information, if correctly validated.

More generally, the voluntary submission of BVAHT films and the non-random sampling of offspring has the potential to affect the genetic trend. As had been previously reported [11], the proportion of parents with BVAHT scores towards the end of the study is quite high. In earlier time periods represented by the study, a much lower proportion of parents had BVAHT scores available and so the average EBVs for earlier years is likely to be less accurate. One might expect that as in Paster et al. [36] favourable scores would be more likely to be reported and the average EBV to have been biased favourably. This would suggest that a genetic improvement has indeed occurred. However, the possibility that earlier data were biased unfavourably, or that later data, despite being much more complete, have been biased favourably, cannot be completely discounted.

A possibility which this paper was unable to explore, due to limitations of the available software, was EBVs obtained through multivariate ordinal analysis. When phenotypes are available from traits genetically correlated with the trait of interest, incorporation of these data into the EBV trait of interest can increase the accuracy of this EBV [37], [38]. While the same software limitation that prevented multivariate ordinal EBVs also prevented ordinal estimations of genetic correlations of BVAHTs, the correlations found between the EBVs for the BVAHTs suggest there are indeed substantial genetic correlations. Therefore, had multivariate ordinal analyses been possible, use of all phenotypic data from each BVAHT simultaneously could have improved accuracy of all the BVAHT EBVs. Multivariate analysis using linear models was, however, possible using the software available and would have allowed, at least theoretically, the use of data from every BVAHT. Such an approach would, however, make unwarranted methodological assumptions relating to the linearity of the data and difficulty with obtaining positive definite dispersion matrices may have limited the number of BVAHTs which could be included in a multivariate model in any case. Ultimately, the authors decided to persist with ordinally derived EBVs, but it remains possible that multivariate linear EBVs may have a better correlation with CHD-related welfare, due to inclusion of more information, despite the methodological shortcomings. This is an important limitation to the methodology used here and further work exploring the possibility of EBVs derived from multivariate analysis, both linear and ordinal, would be worthwhile.

Calculation of EBVs over a cohort with dates of birth scanning decades provided an opportunity to examine the genetic trend of BVAHTs within this population over 25 years, by expressing the trend on the original BVAHT scale, rather than on the liability scale. There is evidence of genetic improvement in Australian GSDs over time for each of the nine BVAHTs. As we have shown these traits to be both heritable and phenotypically variable in this population, achievement of genetic improvement is not a surprising finding, assuming selection has been taking place upon this phenotype, as quantitative genetics theory predicts that a selection differential applied to a trait that is both phenotypically variable and heritable results in a response in the direction of the selection differential. The improvement seen in all nine traits provides good evidence that a selection differential favouring lower BVAHT scores has been applied by breeders managing this population. If the breeders had been using EBVs instead of phenotypic scores, then a greater genetic improvement would have likely resulted from this selection pressure.

As stated above, advantages of selection using EBVs over phenotype-performance selection include improvement in accuracy and ability to calculate EBVs (albeit of lower accuracy) in animals for which phenotypes are not, or not yet, available. As molecular knowledge of hip dysplasia improves and markers are validated in each breed and population, the potential for marker-enhanced EBVs, i.e. EBVs calculated from a combination of phenotypic and marker data from both an animal and its relatives, have the potential to further improve accuracy of selection [39]. The present study shows significant improvements in accuracy by demonstrating that average accuracy from calculated EBVs for BVAHTs often substantially exceeds accuracies expected from phenotype-performance selection in a cohort of Australian GSDs. The correlation between EBVs for each BVAHT showed a possible underlying pattern in genetic correlations which separates the more phenotypically variable “Group 1” traits from the less variable “Group 2” traits. It is possible that this correlation pattern may be due to these groups differentially measuring the animal’s inherent hip laxity (measured to a greater extent by Group 1 traits) and its tendency to develop osteoarthritic change in response to this laxity (measured to a greater extent by Group 2 traits). Further work has been undertaken to examine the genetic correlations of the nine BVAHTs, and the potential for creating a single index, or possibly a dual index representing the two groups of traits, and will be reported in a future paper. A single or dual index would be substantially more viable as a replacement to phenotype-only selection on the combined phenotype, given that selection based on nine EBVs could potentially be too difficult an adjustment, especially given breeders are accustomed to working with a single phenotype.

## Supporting Information

Figure S1 **A Supplement to** **Figure 5** **.** A- Linear EBVs(x) vs Ordinal EBVs (y) for British Veterinary Association Hip Traits and B- fitted regression values (x) vs standardised residuals (y).
(TIF)
