# Supplementary figures and images for: Estimated Breeding Values for Canine Hip Dysplasia Radiographic Traits in a Cohort of Australian German Shepherd Dogs
Source: PLoS One. 2013 Oct 29;8(10):e77470. doi: 10.1371/journal.pone.0077470 (PMC3812223; doi:10.1371/journal.pone.0077470)

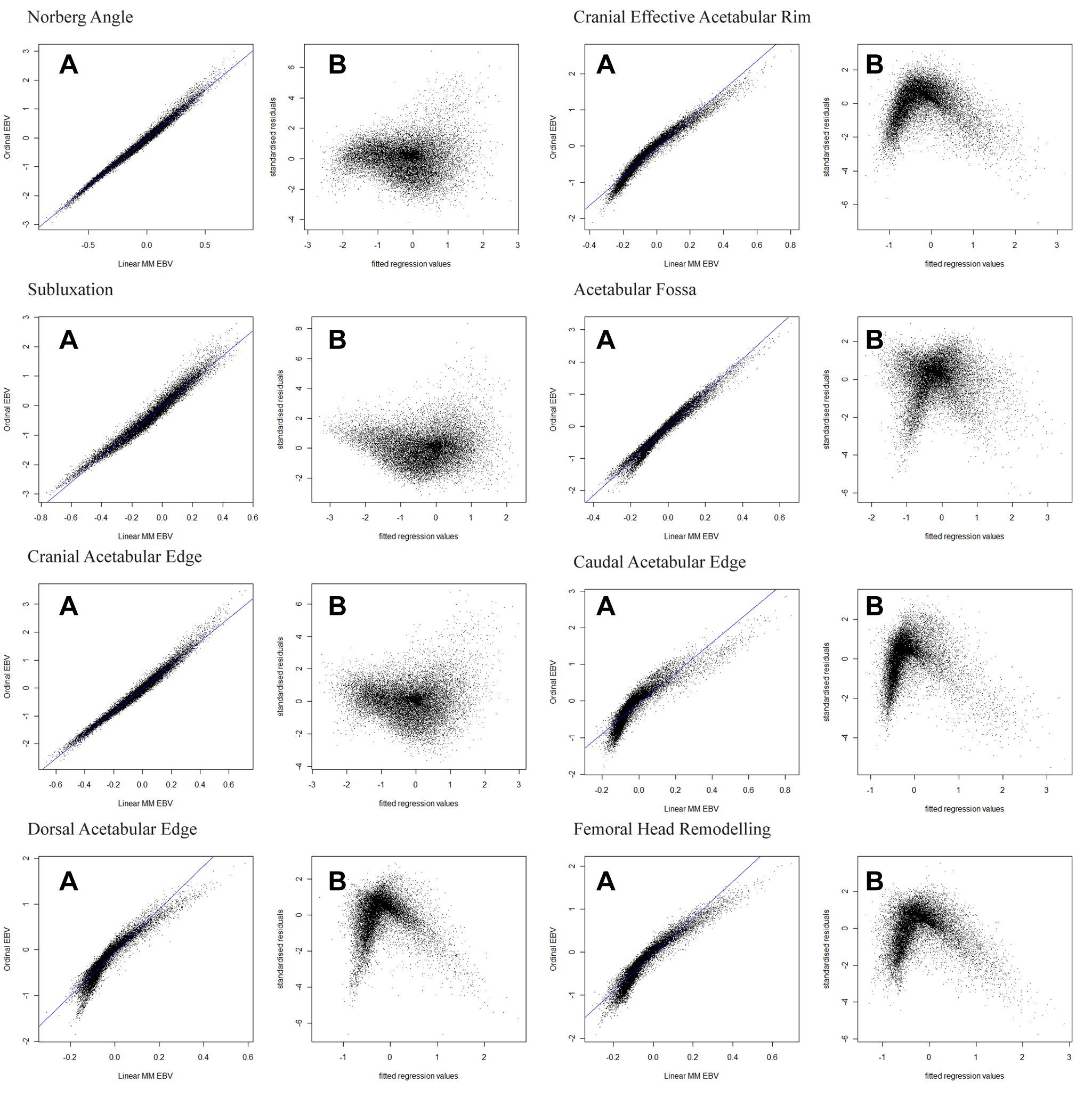

Supplement: Figure S1 — A Supplement to Figure 5 . A- Linear EBVs(x) vs Ordinal EBVs (y) for British Veterinary Association Hip Traits and B- fitted regression values (x) vs standardised residuals (y). (TIF) [file pone.0077470.s001.tif]
